# Sero-prevalence and risk factors associated with occurrence of anti-*Brucella* antibodies among slaughterhouse workers in Uganda

**DOI:** 10.1371/journal.pntd.0012046

**Published:** 2024-03-18

**Authors:** James Katamba Bugeza, Kristina Roesel, Denis Rwabiita Mugizi, Lordrick Alinaitwe, Velma Kivali, Clovice Kankya, Ignacio Moriyon, Elizabeth Anne Jessie Cook

**Affiliations:** 1 International Livestock Research Institute (ILRI), Kampala, Uganda; 2 National Livestock Resources Research Institute (NaLIRRI), Kampala, Uganda; 3 College of Veterinary Medicine, Animal Resources and Biosecurity (CoVAB), Makerere University, Kampala, Uganda; 4 International Livestock Research Institute (ILRI), Nairobi, Kenya; 5 Veterinary Public Health Institute, Vetsuisse faculty, University of Bern, Bern, Switzerland; 6 Institute of Animal Hygiene and Environmental Health, Freie University of Berlin, Berlin, Germany; 7 Departamento Microbiología y Parasitología, Universidad de Navarra, Edificio de Investigación c/Irunlarrea 1, Pamplona, Spain; Institut Pasteur, FRANCE

## Abstract

**Introduction:**

Brucellosis is a febrile zoonosis occurring among high-risk groups such as livestock keepers and abattoir workers and is a public health priority in Uganda. The technical complexities of bacteriological and molecular methods make serological approaches the cornerstone of diagnosis of human brucellosis in resource limited settings. Therefore, proper application and interpretation of serological tests is central to achieve a correct diagnosis.

**Materials and methods:**

We conducted a cross-sectional study to estimate the seroprevalence and factors associated with anti-*Brucella* antibodies among slaughterhouse workers processing ruminants and pigs in three regions of the country with serial testing using a combination of the Rose Bengal Test (RBT) and the BrucellaCapt test. An authorized clinician collected 543 blood samples from consenting abattoir workers as well as attribute medical and social demographic data. Univariable and multivariable logistic regression were used to determine factors associated with anti-*Brucella* sero-positivity.

**Results and discussion:**

The sero-prevalence among ruminant slaughterhouse workers ranged from 7.3% (95% CI: 4.8–10.7) using BrucellaCapt to 9.0% (95% CI: 6.3–12.7) using RBT. Slaughterhouse workers from the Eastern regions (AOR = 9.84, 95%CI 2.27–69.2, p = 0.006) and those who graze animals for alternative income (AOR = 2.36, 95% CI: 1.91–6.63, p = 0.040) were at a higher risk of exposure to *Brucella*. Similarly, those who wore Personal Protective Equipment (AOR = 4.83, 95%CI:1.63–18.0, p = 0.009) and those who slaughter cattle (AOR = 2.12, 95%CI: 1.25–6.0, p = 0.006) were at a higher risk of exposure to *Brucella*. Those who slaughter small ruminants (AOR = 1.54, 95%CI: 1.32–4.01, p = 0.048) were also at a higher risk of exposure to *Brucella*.

**Conclusions and recommendations:**

Our study demonstrates the combined practical application of the RBT and BrucellaCapt in the diagnosis of human brucellosis in endemic settings. Both pharmaceutical (e.g., routine testing and timely therapeutic intervention), and non-pharmaceutical (e.g., higher index of suspicion of brucellosis when investigating fevers of unknown origin and observation of strict abattoir hygiene) countermeasures should be considered for control of the disease in high-risk groups.

## Author summary

Brucellosis is a febrile zoonosis occurring among high-risk groups such as abattoir workers and is a public health priority in Uganda. Whereas bacteriological isolation is conclusive, and some molecular methods have been found useful for diagnosis of brucellosis, they are technically complex and may delay commencement of treatment. Therefore, clinicians from resource poor settings rely on clinical examination and serology for diagnosis of human brucellosis. However, brucellosis lacks pathognomonic signs, and clinically resembles other endemic febrile illnesses which complicates diagnosis. Poor quality *Brucella* antigens, serological tests not validated for human use, and lack of consensus on the tests of choice complicate diagnosis of brucellosis. Previously, many studies employed a variety of tests to estimate sero-prevalence of brucellosis in high-risk groups leading in some cases to over estimation of the disease in Uganda. Here we applied the RBT and BrucellaCapt tests in a serial testing scheme to detect contacts, short and long evolution cases of brucellosis and report a sero-prevalence ranging from 7.3% to 9.0% among slaughterhouse workers in Uganda. *Brucella* seropositivity was associated with the region where participants worked, the slaughter of cattle, small ruminants, and grazing animals as an activity outside the work of the slaughterhouse.

## Introduction

Brucellosis is the name given to a group of highly contagious zoonotic infections caused by members of the genus *Brucella*. Human brucellosis remains one of the most common foodborne or occupational diseases in countries where it is endemic in Latin America, the Middle East, Africa and Asia [1–4]. Brucellosis constitutes a huge economic burden on affected individuals and their families in terms of the costs incurred for hospital diagnosis, treatment, loss of work or income due to illness, and decline in the socioeconomic status from the associated loss of income [1]. Brucellosis is largely a neglected zoonosis and estimates of the annual incidence of the disease is generally lacking making the assessment of the public health impact difficult [2,5].

*B*. *abortus*, *B*. *melitensis* and *B*. *suis* (except for biovar 2) are the main pathogenic species to humans, and *B*. *canis* to a lesser extent [6]. Humans are either infected through consumption of contaminated dairy products, particularly unpasteurized milk, or through broken skin, splashes on mucous membranes and inhalation. Due to their occupation, well-known risk groups are farmers, their families and professionals handling animals, including slaughterhouse workers [7]. Occupational exposure includes handling of abortion materials, butchering and dressing of food animals, and laboratory exposure with transmission occurring through inhalation of aerosolized particles [8,9]. *Brucella* vaccinal strains S19, Rev1 and RB51 can also infect humans through accidental injection during animal vaccinations, through vaccine manufacturing processes, or consumption of raw or undercooked milk from vaccinated animals, since there is a possibility of excretion of vaccinal strains via milk [7,10–12] After a variable incubation period (from a few days to months), *Brucella* can be present in the blood and in a large variety of tissues and organs, causing focal forms and complications, including spondylitis, sacroiliitis, osteomyelitis, orchitis, and neurobrucellosis [6]. Although it occurs in less than 2% of patients, endocarditis is the primary cause of mortality in human brucellosis [13,14].

Clinical examination, patient history, and epidemiological evidence largely form the basis for diagnosis of human brucellosis in resource limited settings. However, clinical diagnosis alone is inadequate because brucellosis signs and symptoms are unspecific, protean and can be confused with those of several other febrile illnesses, including malaria, tuberculosis and typhoid fever, all endemic in many African countries [6,15]. Therefore, it is essential to complement clinical examination with laboratory tests [6,16,17]. A positive bacteriological culture is conclusive and allows identification of the *Brucella* species, but requires appropriate infrastructure, including biosafety, has suboptimal sensitivity and may delay commencement of treatment [17–19]. DNA detection methods are technically too complex for resource limited settings and consensus protocols are lacking [17]. Serological assays are very valuable for diagnosis of human brucellosis and, together with epidemiological evidence and compatible symptoms, can confirm brucellosis cases if correctly interpreted. In the last decade, the Rose Bengal Test (RBT), serum agglutination test (SAT), IgM/IgG lateral flow immunochromatographic assay (LFA), and commercial indirect/competitive-ELISA (i/c-ELISA) have been used for the serodiagnosis of brucellosis in Uganda [20]. However, commercial i/c-ELISA have not been validated for human brucellosis and SAT usefulness is limited by the fact that in brucellosis IgM/IgG/IgA agglutinating antibodies are progressively substituted following infection with IgG/IgA variants that are non-agglutinating [16,21–24]. Thus, while SAT is valuable in acute (i.e., short evolution) cases, when titres are low or doubtful, tests detecting non-agglutinating antibodies are necessary. The later tests include the technically demanding Coombs test, and the so called acid pH agglutination tests, namely the RBT and BrucellaCapt, which detect both acute and long evolution cases [21,25–29]. While the BrucellaCapt uses a microplate format and serum dilutions to obtain a diagnostic titre, the RBT is a rapid agglutination assay well suited for resource limited scenarios. Normally used with plain serum, RBT can be positive in persons from endemic areas and risk groups that do not have clinical symptoms, a problem largely solved by testing serum dilutions [21,30–32]. iELISAs of IgM and IgG specificity are also alternatives but require additional equipment and pose validation issues with regards to the validity of diagnostic cut-offs in different areas [33]. Antibodies induced by some gram-negative bacteria (mainly *Yersinia enterocolitica* serotype O:9) react in all brucellosis tests but, while they may pose a problem in animals, this cross-reactivity is not relevant in human brucellosis because the syndromes caused by such bacteria are clinically very different [34].

Brucellosis is endemic in Uganda and is a priority zoonosis for control [35]. Although important in major food animals in Uganda, particularly cattle and goats [20,36], information about human brucellosis is scanty. According to a widely cited review, the incidence in Uganda is 0.9 cases per million but this estimate is based on incomplete WHO records of the year 2004 [37]. In addition, it is highly probable that this figure reflects records of Ugandan hospitals, which routinely use the febrile *Brucella* antigen (FBAT) kits for diagnosis, a method recently proved unreliable [31,38]. The FBAT recorded poor specificity ranging between 65.2%–75% in a recent study compared to other tests such as RBT, and SAT-Coombs and results do not correlate with exposure [31,38].

Concerning risk groups, using the buffered plate agglutination test (an RBT variant), SAT and c-ELISA, Nasinyama et al. [39] reported 5.8%-9.9% seroprevalence among 329 cattle keepers in Kampala and Mbarara districts. Using RBT and SAT, Tumwine et al. [40] found a sero-prevalence of 17% among 235 livestock keepers in Kiboga (Central Uganda). Following a different test strategy, Ezama et al. and Miller et al. [41,42] reported sero-prevalence of 13.4% and 11% in 214 and 236 livestock keepers in Western and Southwestern Uganda using a combination of IgM LFA/RBT and IgM/IgG LFA, respectively. Although many of the above figures need to be interpreted with caution because of lack of validation (i-/c-ELISAs), potential specificity loss (RBT), inability to detect non-agglutinating antibodies (SAT) or IgG (IgM LFA), they clearly indicate the importance of brucellosis in characteristic risk groups. It is also notable that few or no studies investigated livestock keeping communities of the Northern, Eastern and Northeastern parts of the country.

Abattoir workers are a characteristic risk-group in brucellosis epidemiology and the presence of the disease in this group is an indicator of the public health threat in both humans and animals. Specifically, poor infrastructure and hygiene prevailing in most abattoirs and lack of appropriate personal protective equipment (PPE) make slaughterhouse workers one of the high-risk occupational groups for brucellosis [43,44]. A study conducted by Nabukenya et al. among 232 abattoir workers in Kampala and Mbarara districts found a 10% sero-prevalence using SAT performed either using the standard tube protocol or microplates [45]. Although SAT would detect only recent contacts and acute cases (where non-agglutinating antibodies are not significant), this figure reveals the potential importance of the disease. However, there is no documented evidence of studies conducted to assess the sero-prevalence of anti-*Brucella* antibodies in slaughterhouse workers in Uganda using a serological test strategy capturing both agglutinating and the non-agglutinating (and blocking) antibodies characteristic of individuals that have gone undiagnosed for protracted periods of time. Here we report an estimation of the seroprevalence, and risk factors associated with occurrence of anti-*Brucella* antibodies among slaughterhouse workers in Uganda using serial testing with a combination of RBT and BrucellaCapt test, both of which have been validated and are valuable in the detection of contacts and short and long evolution cases of human brucellosis.

## Materials and methods

### Ethics statement

Written consent was obtained from each of the participants before a health check, interview and sample collection were conducted. Consent forms were translated into the local dialects with the help of native speakers. For participants who could not read and write, the consent form was read to them, and explanations made where necessary, after which they would sign the consent forms by thumb printing. Participants who were evaluated as probable brucellosis cases were referred to the nearest government health facility for management with treatment costs incurred by the research team. To keep the data anonymous participants were assigned unique numbers that were used to identify the samples and metadata and to provide feedback after testing. Those that were evaluated as probable cases of brucellosis were referred to the nearest government health facilities for management. The study was cleared by Makerere University College of Health Sciences Research and Ethics Committee (Ref: MAKSHSREC-2021-116) and the Uganda National Council of Science and Technology (Ref: HS1377ES).

### Study design

A cross sectional study design was adopted for both sample and data collection from workers in three regional slaughterhouses in Uganda between August 2021 and December 2022.

### Study area

**Fig 1:**
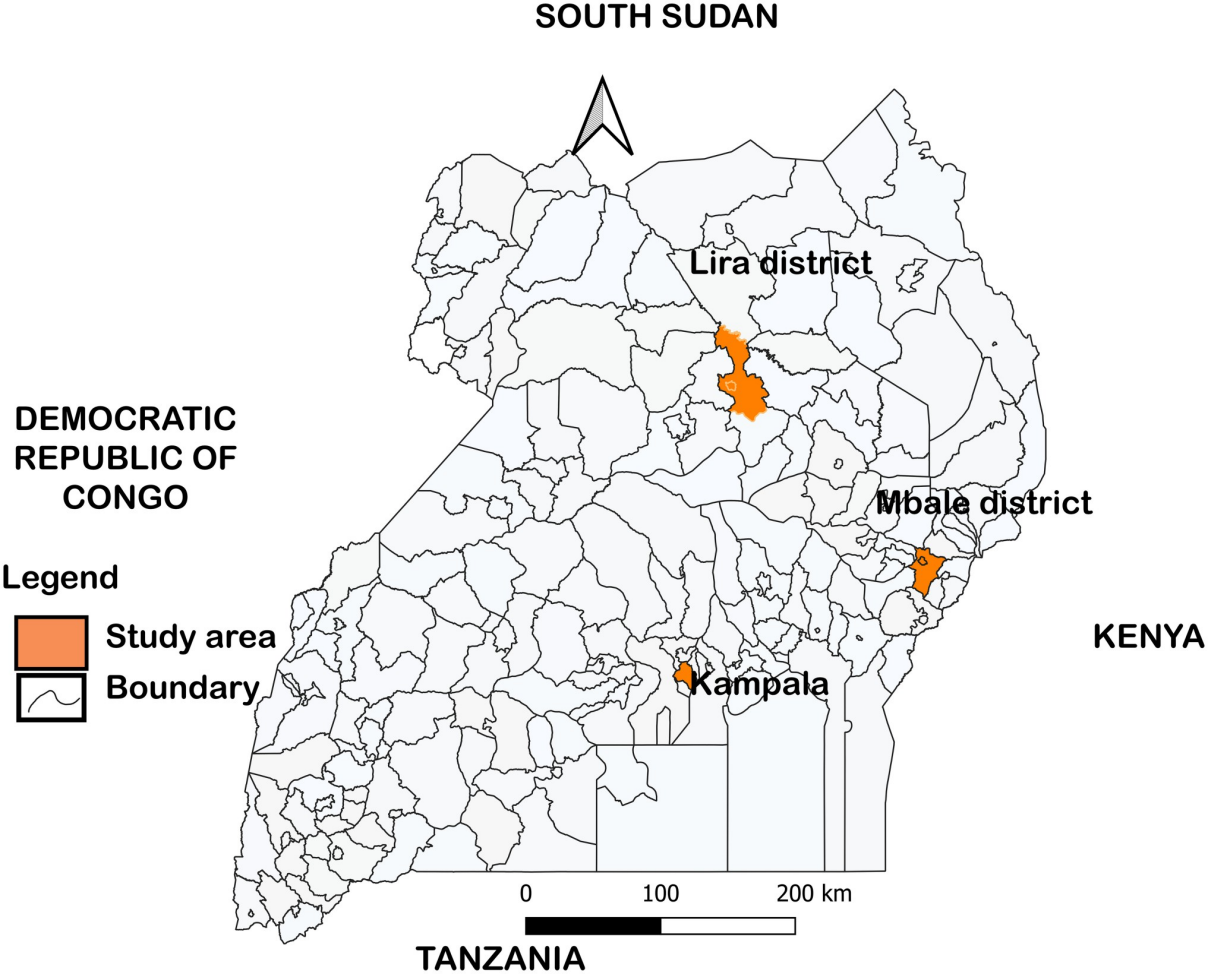
Map of Uganda showing study areas. The shape file for the base map was obtained from https://data.humdata.org/dataset?res_format=SHP&q=uganda&sort=if(gt(last_modified%2Creview_date)%2Clast_modified%2Creview_date)%20desc&ext_page_size=25 and modified by JKB.

The study was conducted in the main ruminant slaughterhouses in Lira District in Northern Uganda, Mbale district in Eastern Uganda and Kampala district in Central Uganda as shown in Fig 1. In Lira district the study was conducted at Lira city abattoir (slaughters both cattle and small ruminants), Omodo Market (slaughters small ruminants) and Teso bar (slaughters pigs). In Mbale the study was conducted at Mbale city abattoir (slaughters both cattle and small ruminants), and in Kampala district the study was conducted at Kampala city abattoir (slaughters both cattle and small ruminants) and Wambizzi abattoir (slaughters pigs). In Lira and Mbale districts, slaughterhouse workers from smaller slaughter slabs were invited and sampled from the main health camps organized at the major slaughter facilities. The map of the study area was developed using QGIS 3.30.1.

### Sample size determination and sample selection

The sample sizes for each region were calculated using the Epitools-epidemiological calculator available at http://epitools.ausvet.com.au. The above method considers the sensitivity and specificity of the diagnostic test, the assumed true prevalence, the precision, and confidence levels. For this study, the sensitivity and specificity of the RBT was assumed to be 100 [21,46]. The confidence level and precision were 0.95 and 0.05, respectively. The assumed prevalence for each region were 18.7% for Northern and Eastern [47] and 17% for Central [40]. Thus, the sample size for each of the North and Eastern regions was 237 participants and 217 for the Central region making a total of 691 blood samples which were targeted for collection.

A one-week health camp featuring a medical tent with a separate phlebotomy area and interview room was organized at each of the slaughterhouses. Every day an appointed person was asked to inform slaughterhouse workers to voluntarily report to the health camp for a general health check-up and to participate in the study at their convenience. Participants were opportunistically recruited into the study upon obtaining written consent. Those who declined to participate, individuals below 18 years, or those not directly involved in the slaughter of animals were excluded.

### Collection of blood samples and sociodemographic data

To collect blood samples, the venepuncture site on the median cubital vein was cleaned with alcohol swabs using back and forth friction scrub and allowed to dry for 30 seconds. Venepuncture was performed by a clinician to obtain about 5ml of blood which was dispensed into clot activation tubes. The participant’s body temperature was taken using an infrared thermometer and other clinical parameters (on a preset checklist) were taken to guide results interpretation. Socio-demographic and relevant epidemiological data was collected from each of the participants and entered in open data kit (ODK) on a tablet. The clot activation tubes were kept at room temperature until clotting was complete. Serum was harvested and dispensed into labelled cryovials which were then kept on ice and transported to the Central Diagnostic laboratory (CDL) at the College of Veterinary Medicine (CoVAB), Makerere University, for serological analysis.

### Screening of serum for anti-*Brucella* antibodies

Sera were screened with the standard RBT (Instituto de Salud Tropical, Universidad de Navarra. www.Istun.es Edificio CIMA. Avda Pio XII, 55.31008, Pamplona, Spain) as described by Diaz et al [21]. Agglutination denoted a positive reaction and vice versa. Positive sera were serially diluted up to a final dilution of 1:32 and retested with RBT following a protocol described by Díaz et al. and Mantur et al. [21,46]. Complementary testing was done using the BrucellaCapt test, performed according to the manufacturer’s instructions (Vircell SL, Granada, Spain) on all RBT positive sera samples. The test is a single step immunocapture agglutination assay and consists of microtiter plates coated with antibodies against human immunoglobulins (IgG and IgA). After the addition and dilution of serum in an acid buffer, the antigen suspension included in the BrucellaCapt kit was added, and the strips sealed. The strips were incubated at 37°C for 24h in a dark, humid chamber. Positive reactions show agglutination over the bottom of the well. Negative reactions are indicated by a pellet at the centre of the bottom of the well [48].

### Data analysis

Sero-prevalence was calculated for both tests by dividing the number testing positive by the number tested (Number positive/Number tested) x100 and confidence intervals were determined using the BinomCI (Jeffrey’s method) command in R version 4.2.2. Bivariable logistic regression using the *glm* function in R was used to screen for factors associated with participants’ seropositivity and p-values of <0.05 were considered significant. A multivariable logistic regression model was fitted using *glm* function in R with backward selection procedure to identify factors associated with participants seropositivity. All variables with p<0.05 and some for which p>0.05 but were biologically plausible were included in the model. The model was evaluated using the Hosmer-Lemeshow goodness of fit test (in the “generalhoslem” package of R). P-values (p ≤ 0.05), Adjusted Odds Ratios (AOR) and 95% confidence intervals were used to determine the factors associated with anti-*Brucella* sero-positivity.

## Results

### Demographic characteristics of participants

Five hundred and forty-three (543) slaughterhouse workers were recruited into the study. Most of the participants (41.4.%) were sampled from the Central region. The average age of the participants was 35 years with range 18 to 78, over 88% of the participants were male and most (51.57%) were involved in the slaughter of cattle. The participants roles in the abattoir included: slaying 22.10%, skinning/dehairing 30.76%, eviscerating 18.42% and decapitating 13.26% among others (Table 1).

**Table 1:** Demographic characteristics of respondents (n = 543).

Variable	Category	Frequency (%)	95% CI
Region	Central	225 (41.44)	37.28–45.72
	Northern	209 (38.49)	34.40–42.74
	Eastern	109 (20.07)	16.83–23.74
Age	18–40	226 (65.7)	60.4–70.6
	41–78	118 (34.3)	29.3–39.6
Abattoir	Kampala City Abattoir	113 (20.81)	17.52–24.52
	Lira City Abattoir	109 (20.07)	16.83–23.74
	Mbale City Abattoir	74 (13.63)	10.91–16.87
	Omodo Market	59 (10.87)	8.44–13.86
	Others	36 (6.63)	4.75–9.15
	Teso Bar	41 (7.55)	5.53–10.19
	Wambizi Abattoir	111 (20.44)	17.18–24.13
Gender	Female	65 (11.97)	9.42–15.07
	Male	478 (88.03)	84.93–90.58
Religion	Christian	397 (73.11)	69.13–76.76
	Muslim	139 (25.60)	22.02–29.53
	Other	7 (1.29)	0.57–2.76
Education level	Primary and below	285 (52.49)	48.19–56.75
	Secondary and above	258 (47.51)	43.25–51.81
Slayer (Halal butcher)	No	423 (77.90)	74.12–81.27
	Yes	120 (22.10)	18.73–25.88
Skinner / Dehairer	No	376 (69.24)	65.14–73.07
	Yes	167 (30.76)	26.93–34.86
Eviscerator	No	443 (81.58)	78.01–84.70
	Yes	100 (18.42)	15.30–21.99
Decapitator	No	471 (86.74)	83.53–89.42
	Yes	72 (13.26)	10.58–16.47
Up hoister	No	482 (88.77)	85.73–91.24
	Yes	61 (11.23)	8.76–14.27
Washing offals	No	502 (92.45)	89.81–94.46
	Yes	41 (7.55)	5.53–10.19
Carcass splitter	No	481 (88.58)	85.53–91.07
	Yes	62 (11.42)	8.93–14.47
Meat cutting	No	393 (72.38)	68.37–76.06
	Yes	150 (27.62)	23.94–31.63
Meat inspector	No	529 (97.42)	95.61–98.53
	Yes	14 (2.58)	1.47–4.39
Live animal transporter	No	509 (93.74)	91.27–95.56
	Yes	34 (6.26)	4.44–8.73
Cleans abattoir	No	538 (99.08)	97.74–99.66
	Yes	5 (0.92)	0.34–2.26
Slaughters cattle	No	263 (48.43)	44.17–52.73
	Yes	280 (51.57)	47.27–55.83
Slaughters small ruminants	No	403 (74.22)	70.28–77.80
	Yes	140 (25.78)	22.20–29.72
Slaughters pigs	No	359 (66.10)	61.75–69.88
	Yes	184 (33.89)	30.12–38.25

### Sero prevalence of anti-*Brucella* antibodies

None of the 184 pig slaughterhouse workers tested positive for anti-*Brucella* antibodies with the RBT and on that basis, they were excluded from further analysis since they were now considered of no further interest to the study. Further scrutiny indicated that 15 workers had been sampled but were not directly involved in slaughter of animals. On serological screening they also turned out to be sero-negative and therefore excluded from further analysis too. Among the ruminant slaughterhouse workers, the sero-prevalence according to results of the standard RBT protocol was 9.0% (31/344) (95% CI: 6.3–12.7). Considering the BrucellaCapt test and a titre ≥ 320 the seroprevalence was 7.3% (25/344) (95% CI: 4.8–10.7) (Table 2).

**Table 2:** Sero-prevalence of anti-Brucella antibodies among ruminant slaughterhouse workers (n = 344).

Test	Positive (n)	Prevalence (95% CI)
RBT	31	9.0 (6.3–12.7)
BrucellaCapt	25	7.3 (4.8–10.7)

The RBT and BrucellaCapt titres and the symptoms in the corresponding persons are presented in Table 3. Out of the 31 sera positive in the standard RBT protocol, eight had a titre ≥ 1:8 in the modified RBT protocol, a very high titre in BrucellaCapt and seven self-reported symptoms (one case did not return this information) compatible with brucellosis. Of the three sera that had an RBT titre of 1:4, two also had a high BrucellaCapt titre and one had a 1:640 titre, just above the proposed titre yielding optimal sensitivity (i.e., 95% for titres ≥ 1:320). The remaining 21 RBT-positive sera had a titre 1:2 (i.e., only agglutinated with the plain serum) and of these only two had a 1:640 titre and 12 had a 1:320 titre with BrucellaCapt. When recorded, symptoms in these 21 slaughterhouse workers were variable.

**Table 3:** RBT and BrucellaCapt titres and symptoms in 31 slaughterhouse workers.

ID	RBT titre	BrucellaCapt titre	Temp (°C)	Other self-reported symptoms
16	1:32	1:5120	37.3	Fever, Vomiting, Headache, Night sweats, Arthralgia, Nausea, Chills
26	1:32	1:5120	36.9	Fever, Vomiting, Diarrhea, Chills
363	1:32	1:5120	37.1	NC
405	1:32	1:5120	37.4	Fever, Known diabetic
298	1:8	1:2560	37.7	Myalgia, Arthralgia
433	1:8	1:5120	35.9	Fever, Headache, Fatigue
443	1:8	1:5120	36.5	Fever, Fatigue, Nausea
266	1:4	1:1280	37.2	Headache, weight loss, loss of appetite, Arthralgia
263	1:4	1:2560	36.8	NC
402	1:4	1:640	36.6	NC
410	1:2	1:640	36.5	Fever, Fatigue, chills
287	1:2	1:640	36.6	Arthralgia
106	1:2	1:320	36.6	Red urine
230	1:2	1:320	36.8	NC
233	1:2	1:320	36.6	NC
239	1:2	1:320	37.2	NC
254	1:2	1:320	36.8	Fever, Arthralgia, swollen joints, Needle prick pain in joints and muscles
277	1:2	1:320	37.1	Fever
280	1:2	1:320	37.3	Fever, cough
297	1:2	1:320	37.2	Myalgia, Back pain
299	1:2	1:320	36.5	NC
452	1:2	1:320	37.2	NC
464	1:2	1:320	36.4	Fever, Headache, Myalgia, chills
475	1:2	1:320	37.1	Fever, Headache
279	1:2	1:160	37.1	Arthralgia
99	1:2	1:80	36.5	NC
235	1:2	1:80	37	Arthralgia, swollen joints
256	1:2	1:80	36.8	Fever
259	1:2	1:80	36.7	NC
349	1:2	1:80	36.2	Headache, abdominal pain
428	1:2	1:80	36.4	Chills
	**31**	**25**		

NC = Not captured: Questions were not filled by the interviewer.

Twenty-two [22] slaughterhouse workers were evaluated as probable brucellosis cases, prescription was obtained from the nearest government health facility, drugs purchased by the research team and patients referred to the same health facilities for management. The therapeutic package consisted of intravenous Gentamicin (160mg O.D x 2/52) and Oral Doxycycline Capsules (100mg B.I.D x 3/52). Complete recovery was reported upon completion of the therapeutic regime in 19 of the patients with 3 registering relapses.

### Summary of participant self-reported symptoms at the time of research

**Fig 2:**
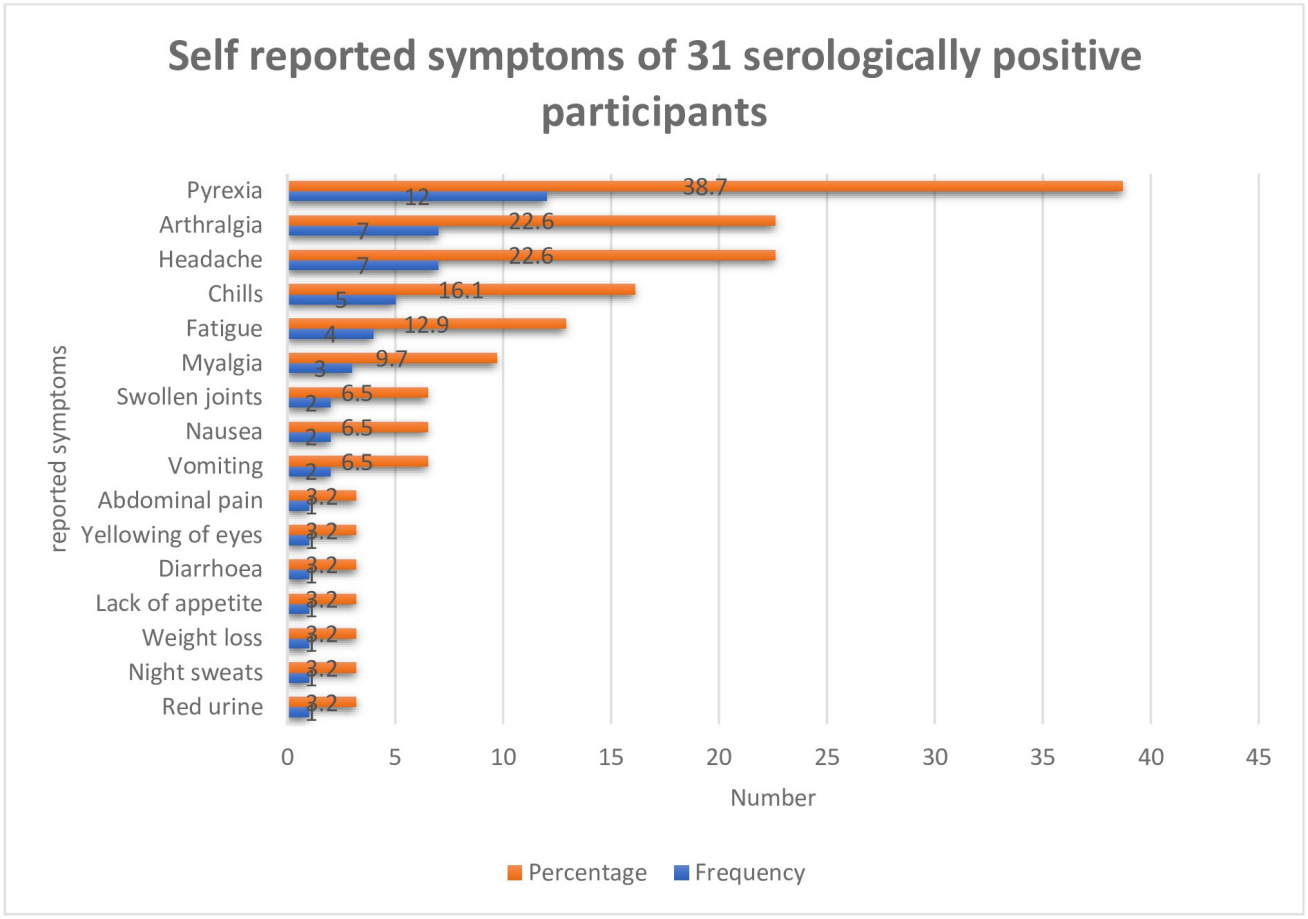
Summary of symptoms reported by serologically positive participants (n = 31).

The symptoms reported by the serologically positive patients were variable but most reported with pyrexia (38.7%), headache (22.6%), arthralgia (22.6%), chills (16.1%), fatigue (12.9%), myalgia (9.7%) and swollen joints (6.5%) (Fig 2).

### Factors associated with anti-*Brucella* sero-positivity

Univariable assessment of ruminant slaughterhouse workers showed that those from the Central region (OR = 6.83, p = 0.014), Eastern region (OR = 12.6, p = 0.001), those who decapitate (OR = 4.48, p = 0.015), those who up hoist carcasses (OR = 4.21, p = 0.038), those who wear PPE (OR = 4.66, p = 0.006) and those who graze/herd animals (OR = 3.13, p = 0.013) were statistically associated with anti-Brucella seropositivity when the BrucellaCapt test results were considered as the outcome (Table 4).

**Table 4:** Univariable analysis of factors associated with anti-Brucella sero-positivity (n = 344).

Variable	Frequency (%)	Positive (%)	OR	95% CI	p-value
**Region**					
Northern^1^	144 (41.9)	2 (1.39)			
Central	114 (33.1)	10 (8.77)	6.83	1.75–45.0	0.014*
Eastern	86 (25.0)	13 (15.12)	12.6	3.38–82.3	0.001*
**Age**					
18 to 40^1^	225 (65.4)	12 (5.33)	—	—	
41and above	119 (34.6)	13 (10.92)	2.18	0.96–5.00	0.062
**Gender**					
Female^1^	34 (9.9)	2 (5.88)	—	—	
Male	310 (90.1)	23 (7.42)	1.28	0.36–8.22	0.744
**Religion**					
Christian^1^	208 (60.5)	12 (5.77)	—	—	
Muslim	136 (39.5)	13 (9.56)	1.73	0.76–3.96	0.19
**Education level**					
Primary and below^1^	194 (56.4)	18 (9.28)	—	—	
Secondary and above	150 (43.6)	7 (4.67)	0.48	0.18–1.13	0.109
**Slayer (Halal butcher)**					
No^1^	311 (90.4)	22 (7.07)	—	—	
Yes	33 (9.6)	3 (9.09)	1.31	0.30–4.08	0.672
**Skinner/ Dehairer**					
No^1^	252 (73.3)	17 (6.75)	—	—	
Yes	92 (26.7)	8 (8.70)	1.32	0.52–3.08	0.539
**Eviscerator**					
No^1^	301 (87.5)	19 (6.31)	—	—	
Yes	43 (12.5)	6 (13.95)	2.41	0.83–6.11	0.079
**Decapitator**					
No^1^	327 (95.1)	21 (6.42)	—	—	
Yes	17 (4.9)	4 (23.53)	4.48	1.18–14.0	0.015*
**Up hoister (carcass raising)**					
No^1^	331 (96.2)	22 (6.65)	—	—	
Yes	13 (3.8)	3 (23.08)	4.21	0.90–15.0	0.038*
**Washes offals**					
No^1^	311 (90.4)	22 (7.07)	—	—	
Yes	33 (9.6)	3 (9.09)	1.31	0.30–4.08	0.672
**Meat cutter**					
No^1^	270 (78.5)	20 (7.41)	—	—	
Yes	74 (21.5)	5 (6.76)	0.91	0.29–2.33	0.849
**Meat inspector**					
No^1^	332 (96.5)	24 (7.23)	—	—	
Yes	12 (3.5)	1 (8.33)	1.17	0.06–6.39	0.885
**Meat transporter**					
No^1^	324 (94.2)	24 (7.41)	—	—	
Yes	20 (5.8)	1 (5.00)	0.66	0.04–3.39	0.689
**Cleans abattoir**					
No^1^	340 (98.8)	24 (7.06)	—	—	
Yes	4 (1.2)	1 (25.00)	4.39	0.21–35.8	0.208
**Slaughters cattle**					
No^1^	67 (19.5)	5 (7.46)	—	—	
Yes	277 (80.5)	20 (7.22)	0.96	0.37–2.99	0.945
**Slaughters small ruminants**					
No^1^	210 (61.0)	15 (7.14)	—	—	
Yes	134 (39.0)	11 (8.21)	1.05	0.44–2.38	0.911
**Wears PPE (apron/overall, boots)**					
No^1^	154 (44.8)	4 (2.60)	—	—	
Yes	190 (55.2)	21 (11.05)	4.66	1.73–16.2	0.006*
**How often cleans apron**					
Every time^1^	197 (57.3)	19 (9.64)	—	—	
Never	147 (42.7)	6 (4.08)	0.4	0.14–0.97	0.056
**How often cleans gumboots**					
Every time^1^	225 (65.4)	21 (9.33)	—	—	
Never	119 (34.6)	4 (3.36)	0.34	0.10–0.91	0.052
**Animal blood splashed in eyes**					
No^1^	240 (69.8)	18 (7.50)	—	—	
Yes	104 (30.2)	7 (6.73)	0.89	0.34–2.12	0.801
**Cut on hands or legs or head**					
No^1^	143 (41.6)	9 (6.29)	—	—	
Yes	201 (58.4)	16 (7.96)	1.29	0.56–3.12	0.558
**Washes hands before handling carcass meat animal parts**					
No^1^	207 (60.2)	17 (8.21)	—	—	
Yes	137 (39.8)	8 (5.84)	0.69	0.28–1.61	0.409
**Washes hands before eating**					
No^1^	242 (70.3)	18 (7.44)	—	—	
Yes	102 (29.7)	7 (6.86)	0.92	0.35–2.18	0.851
**Washes hands following an injury**					
No^1^	319 (92.7)	23 (7.21)	—	—	
Yes	25 (7.3)	2 (8.00)	1.12	0.17–4.12	0.884
**Rears cattle at home**					
No^1^	257 (74.7)	16 (6.23)	—	—	
Yes	87 (25.3)	9 (10.34)	1.74	0.71–4.02	0.205
**Rears goats at home**					
No^1^	233 (67.7)	15 (6.44)	—	—	
Yes	111 (32.3)	10 (9.01)	1.44	0.61–3.28	0.393
**Rears sheep at home**					
No^1^	329 (95.6)	24 (7.29)	—	—	
Yes	15 (4.4)	1 (6.67)	0.91	0.05–4.82	0.927
**Ever handled aborted materials**					
No^1^	292 (84.9)	21 (7.19)	—	—	
Yes	52 (15.1)	4 (7.69)	1.08	0.30–2.98	0.898
**Milks cows**					
No^1^	196 (57.0)	14 (7.14)	—	—	
Yes	148 (43.0)	11 (7.43)	1.04	0.45–2.36	0.918
**Grazes /herds animals**					
No^1^	182 (52.9)	7 (3.85)	—	—	
Yes	162 (47.1)	18 (11.11)	3.13	1.32–8.23	0.013*

*Significant predictors of anti-*Brucella* seropositivity at p <0.05

^1^ Reference category

Multivariable analysis showed that slaughterhouse workers from the Eastern region (AOR = 9.84, 95% CI 2.27–69.2, p = 0.006) were more likely to be seropositive compared to those working in Northern Uganda (Table 5). Although not statistically significant, slaughterhouse workers from the Central parts of the country had high odds of being seropositive (AOR = 4.42, 95% CI:1.04–30.7, p = 0.071). Slaughterhouse workers who graze/herd animals as an alternative income generating activity were also more likely to be seropositive (AOR = 2.36, 95% CI: 1.91–6.63, p = 0.040) compared to those who did not. Furthermore, those who wore PPE (AOR = 4.83, 95% CI:1.63–18.0, p = 0.009), those who slaughter cattle (AOR = 2.12, 95% CI: 1.25–6.0, p = 0.006) and those who slaughter small ruminants (AOR = 1.54, 95% CI: 1.32–4.01, p = 0.048) were more likely to be seropositive. Hosmer and Lemeshow goodness of fit test showed X^2^ = 3.9839, df = 8, p-value = 0.858 implying that the data fitted well with the model.

**Table 5:** Multivariable analysis of factors associated with anti-Brucella sero-positivity.

Variable	AOR	95% CI	p-value
**Region**			
Northern^1^	—	—	
Central	4.42	1.04–30.7	0.071
Eastern	9.84	2.27–69.2	0.006*
**Decapitator**			
No^1^	—	—	
Yes	2.65	0.44–13.7	0.257
**Grazing/herding animals**			
No^1^	—	—	
Yes	2.36	1.91–6.63	0.040*
**Wears PPE**			
No^1^	—	—	
Yes	4.83	1.63–18.0	0.009*
**Age**			
18 to 40^1^	—	—	
41 and above	1.01	0.39–2.58	0.981
**Slaughters small ruminants**			
No^1^	—	—	
Yes	1.54	1.32–4.01	0.048*
**Slaughters cattle**			
No^1^	—	—	
Yes	2.12	1.25–6.00	0.006*

*Significant predictors of anti-*Brucella* seropositivity at p <0.05

^1^ Reference category

AOR–Adjusted Odds Ratio

## Discussion

This study estimated the seroprevalence of antibodies to *Brucella* in slaughterhouse workers in three regions of Uganda and identified potential risk factors for exposure. The sero-prevalence among ruminant slaughterhouse workers in this study ranged between 7.3% (95% CI: 4.8–10.7) to 9.0% (95% CI: 6.3–12.7) (Table 2). This implies that almost one in every 10 slaughterhouse workers was exposed to *Brucella*. Of these, serological evidence shows that at least 1 out 3 (12/31), possibly twice as much (25/31), were actively infected (Table 3). This indicates a high public health threat in this category of industry workers. These estimates are slightly lower than what Nabukenya et al. reported in slaughterhouse workers in Kampala and Mbarara in 2013 using a combination of MAT and STAT, but equally underscore the fact that brucellosis constitutes a health threat in this group of industry workers [45]. The MAT and SAT are the same test in two different formats and the higher sero-prevalence estimates in their study could be attributed to the sample size, antigen standardization and the specific population studied. In their study conducted in Bahr el Ghazal region, South Sudan, Madut et al. reported a seroprevalence of 32.1% among 234 slaughterhouse workers [49]. This high seroprevalence could also be attributed to the small sample size but also the c-ELISA has not been validated for use in the diagnosis of human brucellosis. In Tanzania a higher sero prevalence (48.4%) using SAT was reported by Mirambo et al. a figure which may be attributed to poor specificity of the commercial *B*. *abortus*, and *B*. *melitensis* rapid agglutination tests (i.e., febrile antigen tests) used in the study [31,38,50]. While suggestive, it is not possible to conclude that brucellosis is not a problem among pig slaughterhouse workers in Uganda based on our findings. Depending on the biovar, in other parts of the world pigs are an important source of *B*. *suis*. In Argentina for instance *B*. *suis* biovar 1 has been isolated from workers in pig slaughter and pork processing plants [51–53]. On the other hand, *B*. *suis* biovar 2, endemic in wild life in Europe and a cause of outbreaks in domestic pigs is considered non-zoonotic, with very few human cases reported in individuals with predisposing factors [54,55].

Despite reports of a mild seroprevalence by Erume et al. and Bugeza et al., *B*. *suis* has not been isolated in pigs from Uganda before, warranting further investigation into the apparent low risk of contracting brucellosis from pigs in Uganda [34,51,56,57]. Whether the observed lack of pig to human transmission could be due to exposure of pigs to *B*. *suis* biovar 2 remains a question for future research.

The most reported clinical signs in the seropositive individuals were fever, headache, and arthralgia (Fig 2). These were similar to those observed among 101 patients attending Kabale Regional Referral Hospital, South Western Uganda from September 2002 to May 2010 who were diagnosed with a combination of brucellosis and other co-morbidities [9]. Brucellosis is a complex zoonotic disease lacking specific symptoms, making it easy to confuse with fevers of unknown origin (FUO) and because of the low index of suspicion by clinicians it often goes undetected and untreated [9,30,58]. Positive results also need to be carefully evaluated because antibodies to *Brucella* may result from contact without clinical disease and may remain after successful therapy.

The region of origin, slaughtering cattle, slaughtering small ruminants, and grazing/herding animals as an alternative income generating activity were associated with brucellosis seropositivity (Tables 4 and 5). The high likelihood of slaughterhouse workers from the eastern and central parts of the country being seropositive corelates with the high prevalence in cattle and small ruminants in those areas [36,42,59,60]. Because a large part of the Northeastern region of Uganda is very remote, there is lack of adequate veterinary care [57,61]. This is compounded by communal grazing and transhumant movement of herds in search of water and pastures which facilitates mixing of herds and dissemination of *Brucella* [6,62]. These factors possibly account for the persistence of brucellosis in herds. Therefore, slaughtering animals from these regions may expose slaughterhouse workers to *Brucella*. Previously Madut et al attributed the high sero-prevalence of anti-*Brucella* antibodies in slaughterhouse workers in Wau state, South Sudan, to the large number of animals slaughtered there compared to other regions but perhaps the place of origin of the slaughtered animals had a higher burden of infection [49]. Our model indicated that wearing personal protective equipment (PPE) is one of the factors associated with anti-*Brucella* seropositivity. On the contrary, Nabukenya et al indicated that not wearing PPE was an exposure factors for brucellosis in slaughterhouse workers [45]. Without appropriate PPE, slaughterhouse workers are exposed to *Brucella* if animals are infected. The fact that slaughterhouse workers who wear PPE in our study had higher odds of being seropositive raises concerns on the appropriateness and protectiveness of the PPE against infectious agents like *Brucella*. The PPE observed at the sampled slaughterhouses included just an apron/overall and gumboots. This PPE cannot protect workers from contamination of open wounds or inhalation of aerosols. In addition, workers do not have belts to hold their knives, but instead stick sharp blood stained (infectious) knives in their boots where they meet the bare skin and can potentially injure and contaminate the victim. Also, whether participants wore PPE or not, was self-reported, with a potential bias on the observed results. Public health authorities in Uganda must define and enforce the wearing of appropriate and affordable PPE suitable for the local context.

The slaughter process in all abattoirs visited was highly specialized, with each worker performing a particular task. Examples of slaughter tasks included decapitation, flaying, eviscerating, carcass splitting, up hoisting, washing offals, splitting of heads, etc. Our model did not reveal which tasks were more associated with anti-*Brucella* seropositivity. However, slaughtering cattle and small ruminants were associated with increased odds of being seropositive, and this is correlated with the high prevalence of brucellosis in these animals and confirms active transmission of *Brucella* in occupational settings in Uganda [57]. Mugizi et al. earlier discovered *B*.*abortus* biovars 1, 3 and 7 from cattle samples while a *B*.*melitensis* isolate without biovar assignment was also earlier discovered in Uganda, findings which further support the high likelihood of zoonotic transmission under occupational settings like slaughterhouses [63,64]. The findings also suggest that slaughterhouse workers can be infected outside slaughterhouse through alternative income generating activities like cattle/small ruminant rearing. These activities inevitably bring the individuals into close contact with infectious materials particularly where brucellosis is endemic with limited or no practice of on farm biosecurity. Grazers typically participate in either milking of their livestock, or handling after births while some consume unpasteurized milk and may exposed to infectious material if appropriate PPE is not used or other biosecurity measures are not observed [6,7].

Serological tests remain the cornerstone for diagnosis of the human disease. However, there is a variety of tests, making it essential to choose those more adequate for a given context, and all tests lose some specificity in endemic areas and risk groups so that their interpretation requires careful consideration by an experienced clinician. Provided antigen quality is good, the standard RBT shows excellent performance [21,30,46] and the specificity in persons from endemic areas and risk groups can be optimized by testing serum dilutions. In previous studies RBT resulted in 87%-100% sensitivity-specificity at a ≥ 1:4 [21] or 74%-100% sensitivity-specificity at ≥1:8 [21,46]. While these cut-offs discriminated contacts with 100% specificity, one study [21] recommended that in cases with limited or lower RBT titers and symptoms compatible with brucellosis, BrucellaCapt should be used as a complementary test. Considering that for this test, ≥ 1:160–320 diagnostic titers are recommended [29,48], comparison of the results of this study strongly suggest that a RBT titer ≥ 1:4 is diagnostic under local conditions, making unnecessary a significant proportion of additional tests. However, detailed clinical studies and follow up after antibiotic therapy would be necessary to evaluate the significance of 1:160–320 BrucellaCapt titers, as all brucellosis tests lose some specificity in endemic areas and risk groups. However, despite their usefulness, the results of serological tests do not inform about the *Brucella* species.

## Conclusions and recommendations

Our study highlights the challenges of serological testing and demonstrates the practical application of the RBT and BrucellaCapt in the diagnosis of human brucellosis in endemic settings, a simple strategy that requires little infrastructure and avoids some of the sensitivity problems of standard serum agglutination test and validation issues of iELISAs. There is a high prevalence of anti-*Brucella* antibodies among slaughterhouse workers in Uganda with the Eastern and Central parts of the country most affected. Cattle and small ruminant slaughterhouse workers, and those who engage in animal rearing are more at risk of contracting brucellosis, a finding that confirms active transmission of *Brucella* from both cattle and small ruminants to humans. However, there is need to validate the cut off points of these 2 tests in the context of Uganda using positive and negative controls in addition to isolation of *Brucella* from patient specimens in future studies.

Both pharmaceutical and non-pharmaceutical countermeasures should be considered for control of the disease in high-risk groups. Pharmaceutical counter measures should include routine testing using quality antigens obtained from reference laboratories and timely therapeutic interventions. Non pharmaceutical countermeasures should include awareness raising for both clinicians to be able to raise a high index of suspicion for brucellosis when investigating FUO and for the slaughterhouse workers to implement infection prevention and control measures including implementation of protocols for abattoir hygiene, safe slaughter, donning appropriate PPE, provision of adequate water and soap, first aid kits and infrastructural improvement. The Public Health (Meat) rules statutory instrument 281–18 of the republic of Uganda should be amended to include minimum hygienic standards for slaughterhouses, appropriate PPE and penalties for noncompliance, absence of which makes enforcement impossible.
